# Unveiling the self-association and desolvation in crystal nucleation

**DOI:** 10.1107/S2052252521003882

**Published:** 2021-04-30

**Authors:** Danning Li, Yongli Wang, Shuyi Zong, Na Wang, Xin Li, Yuyuan Dong, Ting Wang, Xin Huang, Hongxun Hao

**Affiliations:** a National Engineering Research Center of Industrial Crystallization Technology, School of Chemical Engineering and Technology, Tianjin university, Tianjin 300072, People’s Republic of China; b Collaborative Innovation Center of Chemical Science and Engineering, Tianjin 300072, People’s Republic of China

**Keywords:** nucleation kinetics, self-association, desolvation, supramolecular structure, crystal engineering, hydrogen bonding, density functional theory, molecular crystals

## Abstract

As the first step in the crystallization process, nucleation has been studied by many researchers. In this work, phenacetin (PHEN) was selected as a model compound to investigate the relationship between the solvent and nucleation kinetics. Induction times at different supersaturation in six solvents were measured. FTIR and NMR spectroscopy were employed to explore the solvent–solute interactions and the self-association properties in solution. Density functional theory (DFT) was adopted to evaluate the strength of solute–solvent interactions and the molecular conformations in different solvents. Based on these spectroscopy data, molecular simulation and nucleation kinetic results, a comprehensive understanding of the relationship between molecular structure, crystal structure, solution chemistry and nucleation dynamics is discussed. Both the solute–solvent interaction strength and the supramolecular structure formed by the self-association of solute molecules affect the nucleation rate. The findings reported here shed new light on the molecular mechanism of nucleation in solution.

## Abstract 2

The importance of self-association and desolvation in the process of crystal nucleation is emphasized; the rearrangement of supramolecular structures also has a non-negligible effect on the nucleation kinetics.

## 1. Introduction   

Crystallization from solution is one of the most widely used unit operations and has been extensively used in the food, chemical, dye, pharmaceutical and agrochemical industries. Nucleation is crucial as the first step of the crystallization process. It can affect the physical properties of a crystal, such as the structure, shape, defects, domain size and polymorphs (Ou *et al.*, 2020 ▸).

Although there have been more and more studies on clusters in the nucleation process over the past decade, the molecular mechanism of the process is still unclear. During crystal nucleation, molecular aggregates in solution play an important role, though their characterization is extremely difficult to describe. Recent evidence suggests that solution chemistry can be an effective method to investigate self-assembly in the nucleation process by exploring the solute–solvent interactions and self-association of solute molecules in solution (Davey *et al.*, 2013 ▸; Gebauer *et al.*, 2014 ▸; Tang *et al.*, 2018 ▸). For example, Davey and Trout used FTIR to study the existing form of butynoic acid in different solvents. In chloroform it is a carboxyl hydrogen-bonded dimer, whereas in ethanol, though the solute has the potential to form hydrogen bonds, it does not participate in the formation of dimers. This result also matches the crystallization behaviour. Dimers are easily formed in non-polar solvents and cyclic structures are easily formed in polar solvents (Parveen *et al.*, 2005 ▸; Chen & Trout, 2008 ▸). Other systems were investigated using the solution chemistry method, such as 2,6-dihydroxybenzoic acid (Davey *et al.*, 2001 ▸), *p*-acetanisidide (Saito *et al.*, 2002 ▸), isonicotinamid (Kulkarni *et al.*, 2012 ▸; Maggioni *et al.*, 2017 ▸), benzoic acid (Tang, Zhang *et al.*, 2017; Burton *et al.*, 2010 ▸), tolfenamic acid (Tang, Mo *et al.*, 2017; Mattei & Li, 2012 ▸), mandelic acids (Davey *et al.*, 2006 ▸), inosine (Chiarella *et al.*, 2007 ▸), *etc.* Recent advances in computational and analytical techniques have also facilitated investigations of larger molecular clusters more efficiently (Sosso *et al.*, 2016 ▸). Therefore, several attempts have been made to explain the evolution of the so-called ‘growth unit’ during nucleation (Gavezzotti *et al.*, 1997 ▸; Di Tommaso, 2013 ▸; Zeglinski *et al.*, 2018). By studying the molecular structure evolution pathways from solute molecules to supramolecular arrangements, the structure correlation between the solution aggregates and crystal syntheses can be explained (Byrn *et al.*, 1976 ▸; Bernstein & Hagler, 1978 ▸; Habgood, 2012 ▸).

From these explorations it was found that not only can the final crystal structure and nucleation mechanism be determined by the solvent, but that the nucleation rate can also be affected by the solvent in the crystallization process. Therefore, a number of researchers suggested a link between the solution structure and measured nucleation kinetic data (Davey *et al.*, 2015 ▸; Sullivan *et al.*, 2014 ▸). For example, Davey *et al.* studied the influence of solvent on nucleation kinetics and concluded that the process of solute dimerization and desolvation is the speed-control step of the entire nucleation process (Sullivan *et al.*, 2014 ▸). Similar conclusions have also been obtained in other systems, such as risperidone, where the stronger the solvent–solute interaction is, the slower the nucleation rate will be (Khamar *et al.*, 2014 ▸; Mealey *et al.*, 2015 ▸). However, other research studies showed contrasting results, for example, a series of structure-related benzoic acids were investigated in four solvents. For a variety of solvents and solutes, this assumption was still valid, but when all the solutes and solvents were considered, it did not hold, and eventually led to the aromatic stacking being assigned the key step in nucleation (Cruz-Cabeza *et al.*, 2017 ▸).

**Figure 1:**
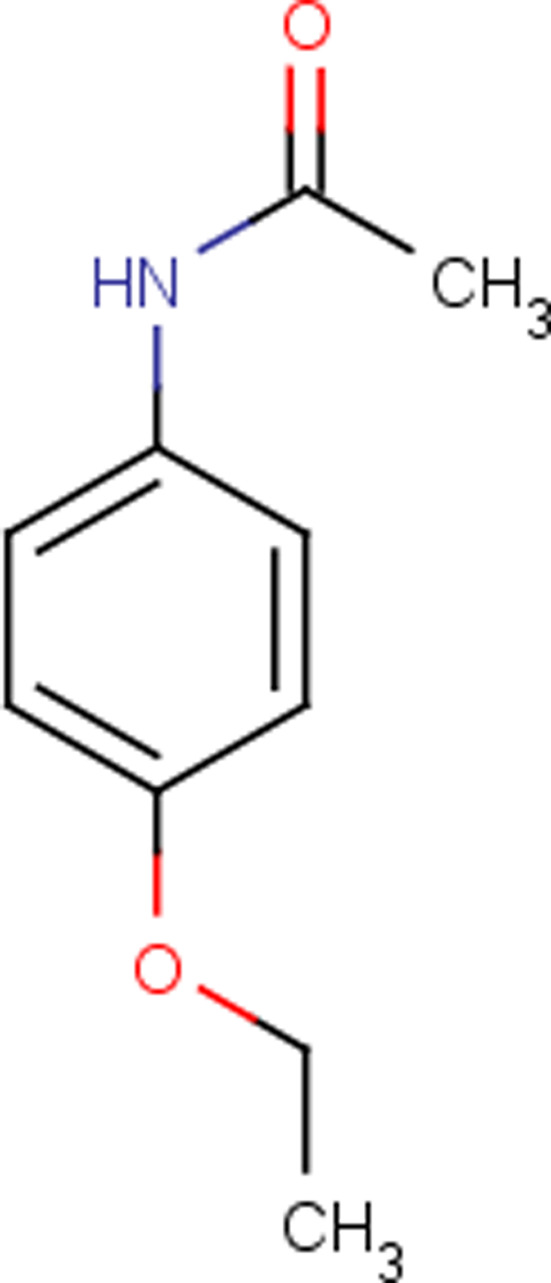
Chemical structure of phenacetin (PHEN).

In order to better understand the relationship between molecular conformations, crystal structure, solution chemistry and nucleation kinetics, investigations focused on the relationship between solution chemistry and nucleation kinetics were carried out here using phenacetin (PHEN) as the model compound, as shown in Fig. 1 ▸. The nucleation process of PHEN in six solvents [chloro­form, aceto­nitrile, methanol, toluene, *N*,*N*-di­methyl­acetamide (DMA), di­methyl sulfoxide (DMSO)] was investigated through a combination of spectroscopic techniques (FTIR, NMR, NOESY) and computational methods [density functional theory (DFT)]. Crystallization of PHEN was carried out in six solvents, and the induction time under different supersaturations was measured to obtain the nucleation kinetic data. Furthermore, computational chemistry was also employed to aid in consistent interpretation, linking solute–solvent interactions and molecular conformations to nucleation behaviours.

## 2. Experimental and simulation methods   

### 2.1. Materials   

Phenacetin (PHEN) was purchased from Shanghai Yuanye Biological Technology Co. Ltd, China, and its mass fraction purity was higher than 98%. All the solvents employed (methanol, chloro­form, aceto­nitrile, toluene, DMA and DMSO) were analytical reagent grade with molar purity higher than 99.5% and were obtained from Tianjin Kewei Chemical Technology Co. Ltd, China. Chloro­form-*d* (99.8% D), aceto­nitrile-*d*_3_ (99.8% D), methanol-*d*_4_ (99.8% D) and DMSO-*d*_6_ (99.8% D) were purchased from SAAN Chemical Technology Co. Ltd of China. All chemicals were used without any further purification.

### 2.2. Induction time measurement   

The solubility data for PHEN were collected using the gravimetric method (equilibrium for 24 h, three repeats) at 25 and 40°C, and are presented in Table S1 of the supporting information. For the purpose of solubility measurement, data were employed to give guidance for the concentration of detection in ATR-FTIR (attenuated total reflectance Fourier transform infrared spectroscopy) and prepare a solution with different concentrations to create specific supersaturations. The definition of the supersaturation ratio is *S* = *x*/x*, where *x* is the actual mole fraction of PHEN and x* is the equilibrium solubility mole fraction.

The induction time, which is defined as the time when the constant supersaturation is established (temperature of solution reaches nucleation temperature) to the moments at which the detectable crystal particles appear. The turbidimeter (Crystal Eyes, DMS-2, HEL Ltd) was used to monitor the formation of nuclei. To begin this process, a round-bottomed jacketed glass batch crystallizer (350 ml) was employed to prepare different concentrations of solution by dissolving appropriate amounts of PHEN in the respective solvents at 45°C. To stir thoroughly, a mechanical stirrer with an agitation speed of 300 rpm was used in the crystallizer. The temperature was controlled by two thermostats (Xianou Laboratory Instrument Works Co. Ltd, Nanjing) connected to two t-branch pipes. The temperature accuracy was ±0.01 K. The temperature was first set at 45°C to dissolve the solid completely, then the t-branch pipes were changed to facilitate shock cooling to 25°C. The point at which the system dropped to 25°C was noted as the start of induction time, and when the turbidmeter indicated a sudden increase this was noted as the end point. In order to reduce the experimental error, six reproducible experiments were performed at each composition.

The relationship between the nucleation rate and supersaturation can be described well using the classical nucleation theory model:
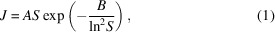


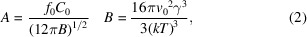
where *J* is the nucleation rate (m^−3^s^−1^), *A* is nucleation kinetic parameter, *S* is the degree of supersaturation, *B* is the nucleation thermodynamic parameter, *f*_0_ is the collision frequency factor independent of supersaturation, *C*_0_ is the concentration of nucleation sites, *v*_0_ is the volume of the solute molecule (m^3^), γ is the interface energy (mJ m^−2^), *k* is Boltzmann constant (J K^−1^) and *T* is the absolute temperature (K). So there is a linear relationship between ln(*J*/*S*) and 1/ln^2^ *s*. By plotting a linear fit, we can obtain the pre-exponential kinetic factor *A* from the intercept and the thermodynamic parameter *B* from the slope.

### 2.3. Powder X-ray diffraction analysis   

Crystals were isolated immediately upon the appearance of solids under different conditions. Powder X-ray diffraction (PXRD) was used to identify samples on a Rigaku D/max-2500 (Rigaku) using Cu *K*α radiation (0.15405 nm) in the 2θ range 5–50° and with a scanning speed of 8° min^−1^ to determine the crystal form nucleated in these experiments. The results showed that all the PHEN obtained in this work was pure form I.

### 2.4. Single-crystal growth and crystal structure analysis   

The slow solvent evaporation method was employed to obtain single crystals of PHEN form I. Specific amounts of PHEN solid were dissolved in methanol, and then the solution was transferred to an open beaker with parafilm. A few holes were made on the parafilm to ensure the solution evaporated slowly. The whole system was placed into an oven and kept at 293.15 K. Crystals of PHEN and its solvent of appropriate size for single-crystal X-ray diffraction (SCXRD) were obtained after several days. SCXRD measurements were conducted on a Rigaku Saturn 70 CCD diffractometer using Mo *K*α radiation (λ = 0.71073 Å) with a graphite monochromator. Integration and scaling of intensity data were accomplished using the program *SAINT* (Bruker, 2017 ▸). The structures were solved using the *SHELXS*2014 (Sheldrick, 2014 ▸) suite of programs, and refinement was conducted using *SHELXL*2018 (Sheldrick, 2015 ▸).

### 2.5. FTIR spectroscopy   

Solid spectroscopy data were collected using Bruker ATR-FTIR (attenuated total reflectance Fourier transform infrared spectroscopy), with a resolution value of 4 cm^−1^, a scan time of 32 and wavenumber ranging from 400 to 4000 cm^−1^. An ATR-FTIR spectrometer (ReactIRTM45, Mettler-Toledo) equipped with a Duradisc Dicomp probe was adopted to facilitate solution spectroscopy. For each sample, 32 scans were collected over a spectra range from 650 to 2800 cm^−1^ at 2 cm^−1^ resolution to investigate the molecular structure of PHEN at different concentrations in the six solvents tested. The concentration of PHEN solution used in this work was determined by solubilities in different solvents, which varies from unsaturated to supersaturated.

### 2.6. NMR spectroscopy   

Different concentrations of ^13^C-NMR and ^1^H-NMR spectra were measured in DMSO, aceto­nitrile, methanol and chloro­form. All the ^13^C-NMR and ^1^H-NMR spectra were detected using a 600 MHz liquid NMR spectrometer (Bruker AVANCE III) at 298 K after 32 and 1024 scans. The software *Mestrenova* (http://mestrelab.com/software/mnova/nmr/) was employed to process and analyze the data. The chemical shifts in the ^1^H and ^13^C spectra were determined relative to the internal reference TMS.

### 2.7. Nuclear Overhauser effect spectroscopy   

2D NOESY experiments were carried out for PHEN solution in DMSO, methanol, aceto­nitrile and chloro­form at room temperature using a 600 MHz Bruker AVANCE III NMR spectrometer. 2D NOE spectra were measured with a standard pulse for both F1 and F2 dimensions. The number of F1 increments was 256, each with 65 536 data points in the F2 dimension. The NOE mixing time was optimized to 0.8 s by measuring NOE buildups. The number of scans and dummy scans were set to be 16 and 2, respectively.

### 2.8. Crystal structure analysis   

Hirshfeld surface and 2D fingerprint analyses were employed to quantitatively analyze and compare the intermolecular interactions of PHEN using *Crystal Explorer 17* (Turner *et al.*, 2017 ▸) software.

### 2.9. Computational method   

#### 2.9.1. Potential energy surface computation   

**Figure 2:**
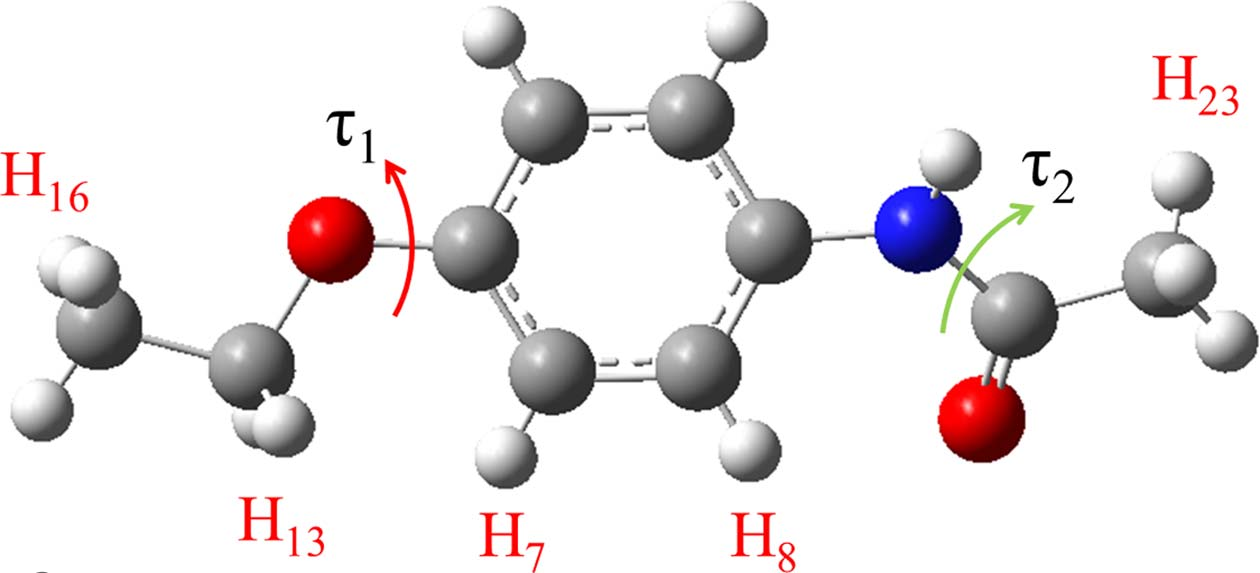
Rotatable single bonds of phenacetin related to conformational change.

For the potential energy surface (PES) scan, the conformer in phenacetin was extracted and its geometry optimized (herein, conformer A). Then the PES of phenacetin conformer A with dihedral angles τ_1_ and τ_2_ (Fig. 2 ▸) was generated by scanning for 18 steps with a step length of 10° for both τ_1_ and τ_2_. All calculations were performed in the gas phase and solvent environment at the M06-2X/631 + G(d,p) level of theory with *Gaussian09* (Frisch *et al.*, 2016 ▸).

#### 2.9.2. Electrostatic potential distribution   

It is generally believed that electrostatic potential can be used to predict and explain the relative molecular orientation and the strength of combination if a complex is mainly assembled by static electricity (such as a hydrogen bond, di­hydrogen bond, halogen bond, *etc*.). The quantitative molecular surface analysis module of the *Multiwfn* program (Lu & Chen, 2012*a* ▸,*b* ▸) is capable of partitioning the whole van der Waals surface into multiple fragments, allowing us to study the characteristics of electrostatic potential distribution (Lu & Manzetti, 2014 ▸). *Multiwfn* and *VMD* (Humphrey *et al.*, 1996 ▸) were used plot the van der Waals surface electrostatic potential distribution.

#### 2.9.3. Solvent–solute interaction calculation   

Density functional theory (DFT) calculations were performed by *Gaussian09* to quantify the interactions in (1:1) molecular complexes of PHEN in the six solvents (Frisch *et al.*, 2016 ▸). The geometries were optimized by the hybrid M06-2x function and 6–31 + G(d,p) basis set with the Grimme D3 dispersion correction using the SMD implicit solvation model (Grimme *et al.*, 2010 ▸; Pratt *et al.*, 2007 ▸). The Grimme dispersion correction allows a good description of weak interactions, such as van der Waals interactions. The binding energy (Δ*E*_bind_) between two molecules is calculated using the following equation:
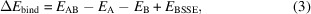
where E_AB_ is the energy of the PHEN–solvent complex, and *E*_A_ and *E*_B_ are the energies of the isolated monomer PHEN and the solvent, respectively. All the energies have been corrected for the zero-point vibrational energies. BSSE is the basis set superposition error and is calculated to correct the over-estimation of binding energies caused by overlapping of the basis functions (Boys & Bernardi, 1970 ▸).

## 3. Results   

### 3.1. Crystallization outcomes   

**Figure 3:**
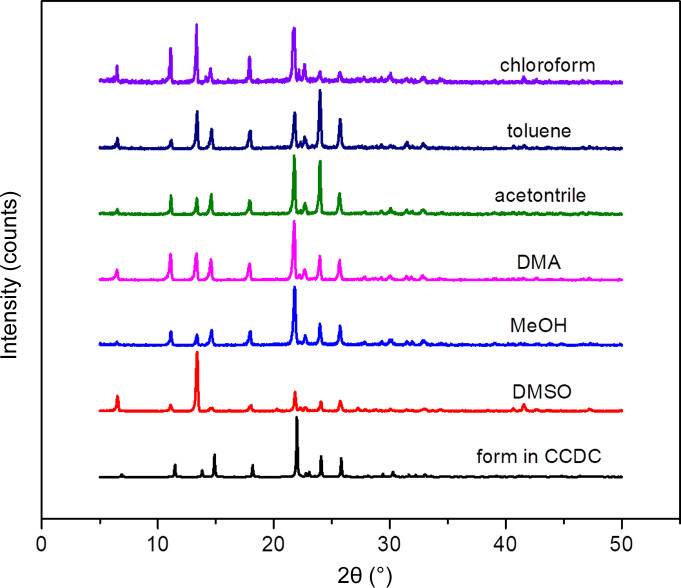
PXRD patterns of PHEN crystallized from different solvents.

The solid forms of PHEN in methanol, chloro­form, aceto­nitrile, toluene, DMA and DMSO at different supersaturations and temperatures were studied. In all solutions form I was obtained and the corresponding PXRD patterns of are shown in Fig. 3 ▸.

The experimentally measured PXRD patterns of PHEN crystallized from different solvents and the simulated patterns from the Cambridge Structural Database (CSD) are compared in Fig. 3 ▸. The main diffraction peaks are consistent, indicating that they are all the same crystal form.

### 3.2. Nucleation rate in different solvents   

The induction time of PHEN in different solvents at various supersaturations was measured and the results are shown in Table S2. Because the volume of solution used to measure the induction time is relatively large (generally ≥ 150 ml), the measured induction time fluctuation does not show the random phenomenon of the induction time usually observed in small volumes. By relating the induction time (*t*) to supersaturation (*S*), it is possible to estimate the nucleation rates and nucleation kinetic parameters of crystals (Zong *et al.*, 2019 ▸).

**Figure 4:**
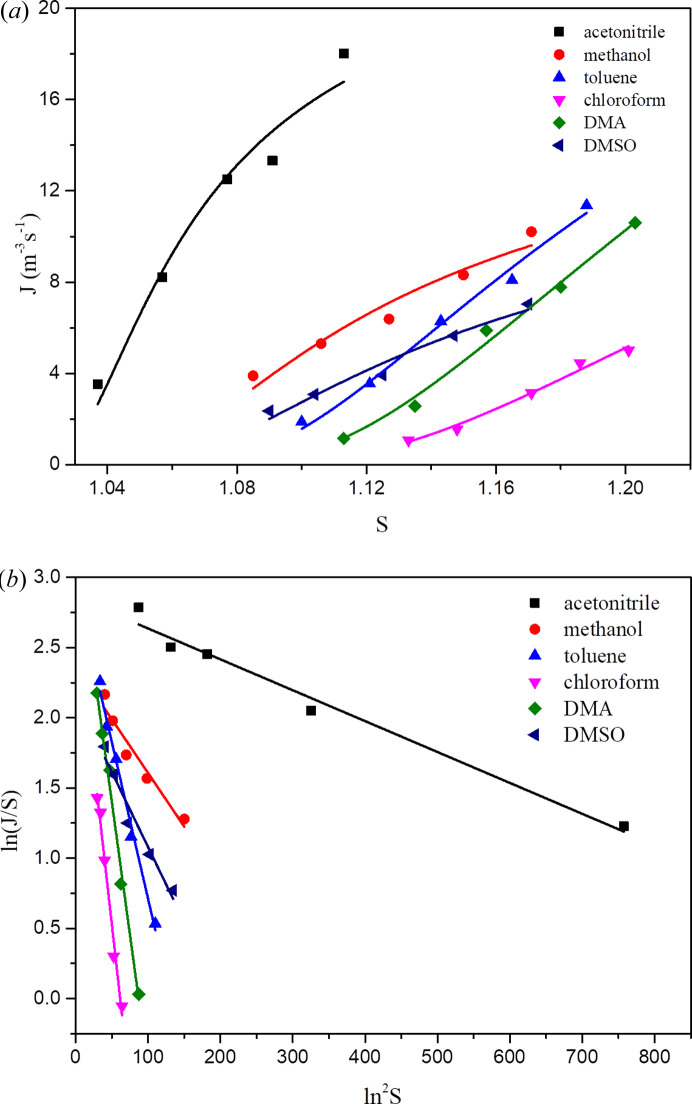
Relationships between (*a*) nucleation rates *J* and supersaturation *S*; (*b*) ln(*J*/*S*) and 1/(ln^2^ *S*) according to classic nucleation theory.

Fig. 4 ▸(*a*) presents the relationship between the nucleation rate *J* and solution supersaturation *S*. The results indicate that PHEN showed the fastest nucleation rate in aceto­nitrile within the experimental supersaturation, followed by methanol. In the case of low supersaturation, the nucleation rate of PHEN in DMSO is faster than that in toluene, whereas the situation is the opposite for high supersaturation. Among the six selected solvents, the slowest nucleation rate is in DMA and chloro­form. In Fig. 4 ▸(*b*), ln(*J*/*S*) and ln^2^ *S* show a good linear relationship in six solvents, indicating that method, supersaturation range and control of experiment conditions to conduct induction time measurements are suitable for this system. The kinetic parameter *A*, thermodynamic parameter *B*, molecular collision frequency *f*_0_ *C*_0_ and interfacial energy γ can be calculated from the slope and intercept, as shown in Table 1 ▸. It can be seen from the data that the interfacial energy followed the order of aceto­nitrile < methanol < DMSO < toluene < DMA < chloro­form, almost the same with that of nucleation rate data. That is, the greater the interface energy, the more difficult it is to nucleate, indicating that nucleation is mainly controlled by thermodynamic processes. In contrast, the order of molecular collision frequency was aceto­nitrile < methanol < DMSO < toluene < DMA < chloro­form. A higher collision frequency should lead to a shorter induction time, but this parameter had no obvious relationship with the order of the nucleation rates. It can be explained that the nucleation rate of the same solute molecule in different solvents may be determined by the interface energy, and the interface energy is closely related to the interactions between the solute molecule and the solvent molecule, such as hydrogen bonding and solvation.

### 3.3. Crystal structure analysis   

The single-crystal data of PHEN form I are presented in Table 2 ▸. According to the SCXRD data, PHEN form I belongs to the monoclinic crystal system and the space group is *P*2_1_/*c*. There is one PHEN molecule in the asymmetric unit.

**Figure 5:**
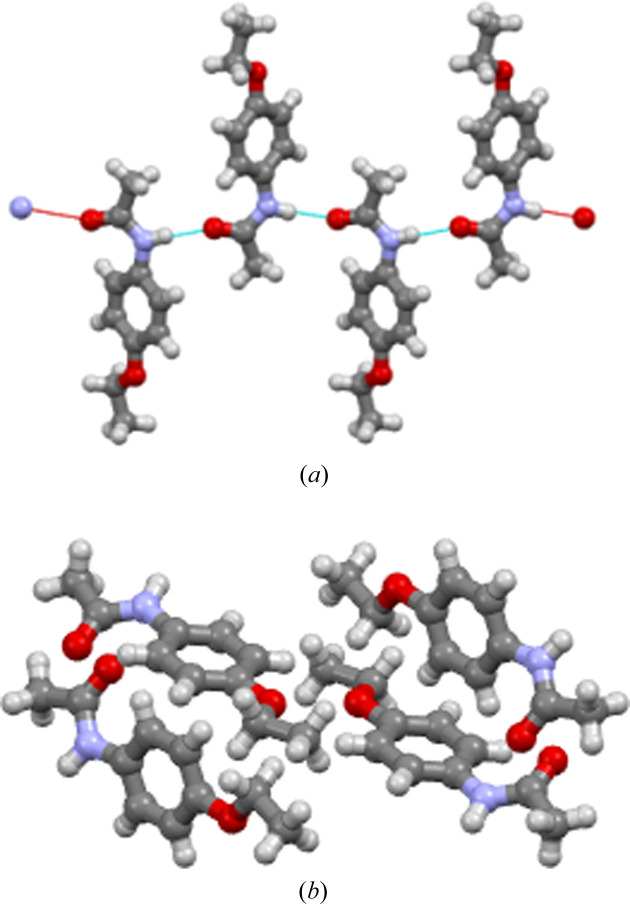
(*a*) Crystal packing of form I along the *c* axis; (*b*) C—H⋯π interaction in form I.

**Figure 6:**
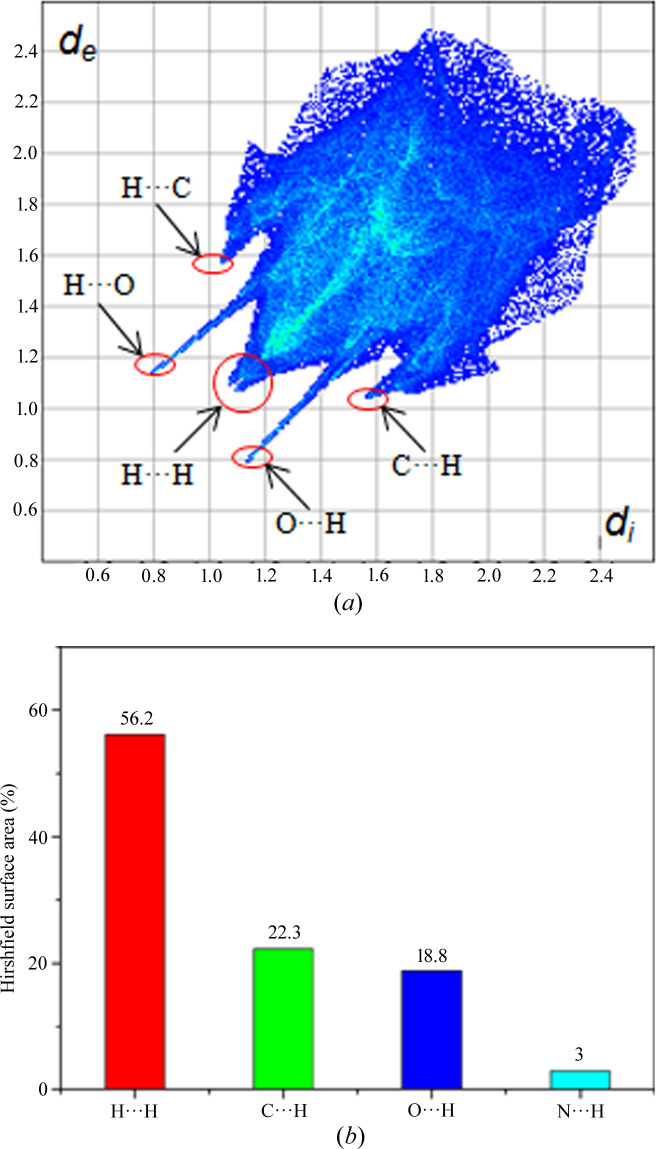
(*a*) 2D fingerprint plot of PHEN form I; (*b*) percentage of various contacts contributing to the Hirshfeld surface area.

The crystal structures of PHEN form I are shown in Fig. 5 ▸. The Hirshfeld surface was further used to quantify the different types of interactions and their contributions in crystal packing. The 2D fingerprint plot and the percentage of various contacts are shown in Fig. 6 ▸. In Fig. 5 ▸(*a*), form I was arranged alternately through N—H⋯O interactions, which corresponds to O⋯H as the strongest interactions in the 2D fingerprint plot. It can be seen that H⋯H contacts and C—H⋯π interactions contribute most to the Hirshfeld surface in form I, which is in agreement with the crystal structure analysis in Fig. 5 ▸. The H⋯H contacts contributing the most part (56.2%) are likely due to the short contacts between the aromatic rings. The formation of a hydrogen bond between the amine and carboxyl group leads to close H⋯H contacts. On the other hand, the hydrogen atoms on aromatic rings become close when aromatic interactions (C—H⋯π and π⋯π) are formed, which contributes another part of the H⋯H contacts.

### 3.4. FTIR spectroscopy   

**Figure 7:**
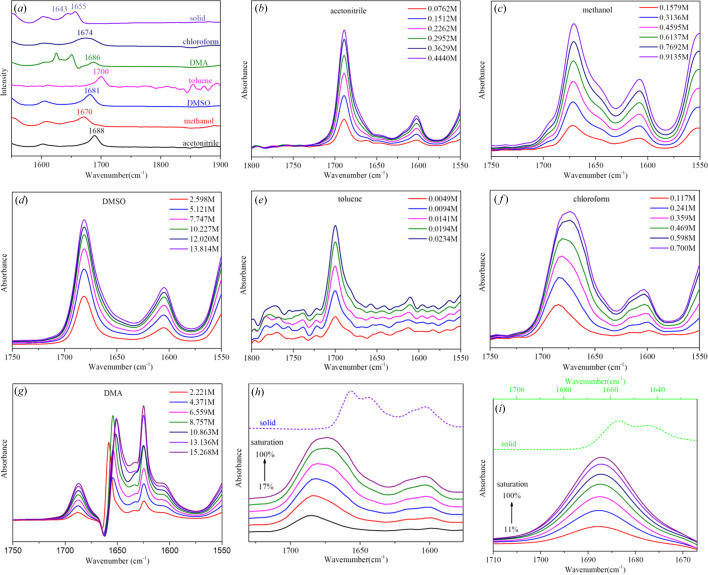
(*a*) IR spectra of PHEN form I and PHEN solutions in different solvents; (*b*)–(*g*) IR spectra of PHEN solutions in aceto­nitrile, methanol, DMSO, toluene, chloro­form and DMA at different concentrations; (*h*) FTIR data of PHEN and PHEN in chloro­form at different concentrations; (*i*) FTIR data of PHEN and PHEN in DMA at different concentrations. The solvent spectrum has been subtracted from all the solution spectra.

The solid spectra of PHEN show strong bands for carbonyl stretching at 1643 and 1655 cm^−1^, indicating the formation of strong hydrogen bonds, as shown in Fig. 7 ▸(*a*). The crystal structure in Fig. 5 ▸(*a*) shows the formation of hydrogen bonds between the carbonyl and the amine group, which is consistent with the results of the solid spectra.

Compared with the solid spectra, the carbonyl peak in the IR spectrum of PHEN in solution shows an obvious blue shift, this indicates the weakening of interactions of the carbonyl groups in the solution. The observed displacements of the ν(C=O) modes upon solution results from the reduction in the C=O⋯H—N hydrogen bonding present between the molecules in solution. The different values in different solvents are associated with the involvement of hydrogen bonding. Thus, it is reasonable to assume that the solute molecules were solvated by the solvents in solution. The stronger the interactions, the more the carbonyl band is displaced to lower wavenumbers. This feature can be used to rank the strength of solvent–solute interactions (Khamar *et al.*, 2014 ▸; Mealey *et al.*, 2015 ▸). Thus, Fig. 7 ▸(*a*) shows that the interaction strength of the PHEN carbonyl with the solvent increases in the order toluene < aceto­nitrile < DMA < DMSO < chloro­form < methanol.

The IR spectra of PHEN in aceto­nitrile, methanol, DMSO and toluene [Figs. 7 ▸(*b*)–7(*e*)] show strong bands for carbonyl stretching at 1688, 1670, 1681 and 1700 cm^−1^, which represents varying degrees of solvation. Aceto­nitrile and DMSO can be hydrogen acceptors, whereas toluene is neither a hydrogen-bond donor nor a hydrogen-bond acceptor. It can only form weak interactions through C—H. Thus, we expect PHEN to show the highest stretching vibration peak of the carbonyl group in toluene. The IR spectrum of PHEN in toluene shows two peaks in the carbonyl group region, the strong peaks at 1700 cm^−1^ indicate the existence of non-solvated aggregates in toluene, and the weaker one at 1680 cm^−1^ suggests a small fraction involve hydrogen bonding. The shoulder peak of low wavenumber in toluene is not obvious due to the too-low solubility of PHEN in toluene. With increasing solute concentration in methanol, the carbonyl peak shows a shoulder at a lower wavenumber about 1655 cm^−1^ which continually increases with concentration. This phenomenon suggests an increase in strong bound carbonyl species with an increase in the concentration of solute. It is difficult to interpret the solute–solute aggregation information in alcohol solutions, as alcohol can act as both a hydrogen bond donor and a hydrogen bond acceptor, which makes it difficult to differentiate solvent–solute and solute–solute interactions.

The IR spectra of PHEN in chloro­form shows that the stretching peak of carbonyl group has a significant red shift with increasing concentration, indicating that the C=O hydrogen-bond complex is formed. According Fig. S1 of the supporting information, the spectra of the same concentration of PHEN in chloro­form and chloro­form-*d* have the same value for carbonyls, indicating that the hydrogen-bond complex is a solute–solute aggregate formed by C=O⋯H—N, which is consistent with the hydrogen bond formed in the crystal. At the same time, as the concentration increases, the blue shift of the benzene ring stretching peak also shows self-association between solutes. This supports that C=O⋯H—N and π⋯π interactions play an important role in self-association. Furthermore, a similar trend was observed in DMA solution: with increasing concentration the spectra move towards their position in the solid state.

### 3.5. NMR spectroscopy   

**Figure 8:**
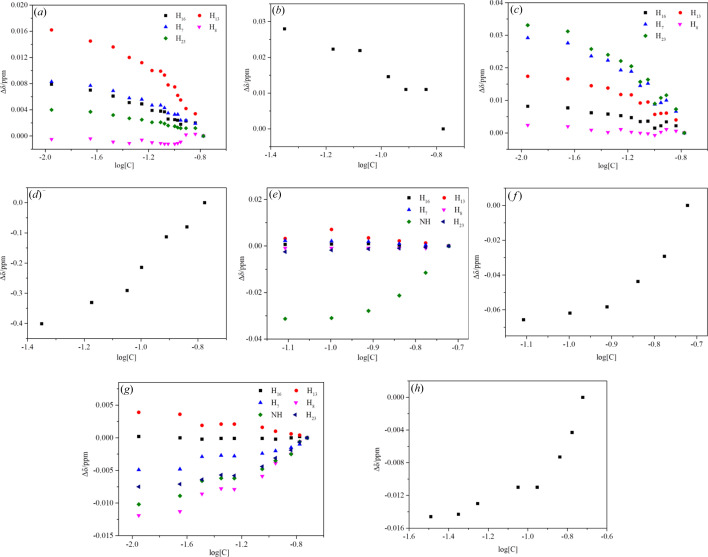
Chemical shifts of ^1^H and ^13^C on the carboxyl group change with concentration at room temperature. (*a*) and (*b*) PHEN in methanol-*d*_4_, (*c*) and (*d*) PHEN in chloro­form-*d*, (*e*) and (*f*) PHEN in aceto­nitrile-*d*_3_, (*g*) and (*h*) PHEN in DMSO-*d*_6_.

^1^H NMR and ^13^C NMR chemical shifts are concentration-dependent in methanol-*d*_4_ [Figs. 8 ▸(*a*)–8(*b*)]. All the protons except H_8_ and the C=O ^13^C display downfield changes when the concentration increases, indicating desolvation. H_13_ show the largest changes, implying the solvation effect is facilitated by hydrogen bonding formed between the eth­oxy on PHEN and the hydroxyl on methanol. As the concentration of PHEN increases, the proportion of solvent decreases and the solvation strength decreases. Chemical shifts of ^1^H and ^13^C in chloro­form-*d* were present in Figs. 8 ▸(*c*) and 8(*d*). All the protons display downfield changes with increasing concentration, which supports desolvation and self-association. H_23_ shows the largest changes, indicating that the carbonyl group plays an important role in the desolvation process. H_7_ exhibits a shielding effect as the concentration increases, these changes can be attributed to self-association through C=O⋯H—N and PHEN–PHEN stacking in the solvent (Tang, Mo *et al.*, 2017 ▸), which also corresponds to the deshielding effect of the carbonyl group; these correspond to the red shift of the carbonyl group and the blue shift of the C=C of the benzene ring in the IR spectrum.

In aceto­nitrile-*d*_3_ [Fig. 8(*f*)], carbonyl ^13^C of PHEN unveils similar concentration-dependent changes to those seen in chloro­form-*d*. The deshielding effect of NH and carbonyl ^13^C can be attributed to self-association through C=O⋯H—N and π⋯π interactions. The chemical shift of ^1^H changed dramatically in DMSO-*d*_6_, and the trend suggests two concentration-dependent events [Figs. 8(*g*) and 8(*h*)]. The increased chemical shift of H_7_, H_8_, H_23_ and NH and the reduced chemical shift of H_13_ are associated with the competitive phenomenon of desolvation and self-association.

### 3.6. 2D NOESY spectra   

**Figure 9:**
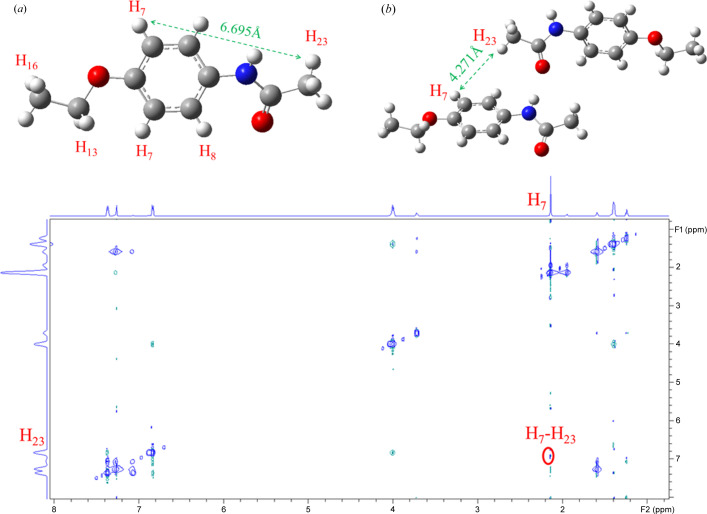
Top: (*a*) distance between H_7_ and H_23_ in a PHEN molecule, (*b*) distance between H_7_ and H_23_ while PHEN molecules assemble by forming a C=O⋯H—N hydrogen bond. Bottom: 2D NOESY of PHEN in chloro­form-*d* at room temperature.

The structural details of these solute–solute and solute–solvent assemblies were further explored by 2D NOESY. As shown in Fig. 9 ▸, the NOE cross peak of H_7_ and H_23_ appears in chloro­form-*d* solution. Combining the crystal structure data in Figs. 9 ▸(*a*) and 9(*b*), the distance between H_7_ and H_23_ is 6.695 Å. But when the PHEN molecules assemble by forming a C=O⋯H—N hydrogen bond, the distance between H_7_ and H_23_ is 4.271 Å. Additionally, an NOE cross peak appears when the space distance is closer than 5 Å. However, PHEN in aceto­nitrile-*d*_3_, methanol-*d*_4_ and DMSO-*d*_6_ did not show the NOE cross peak of H_7_ and H_23_ (Figs. S2 for aceto­nitrile, S3 for DMSO and S4 for methanol). Therefore, PHEN shows an obvious self-association effect in chloro­form, which is consistent with FTIR and NMR spectroscopy results. The self-assembly is not obvious in aceto­nitrile, methanol and DMSO, this may be due to the solvation effect which creates an energy barrier for its self-association.

### 3.7. Molecular conformation   

**Figure 10:**
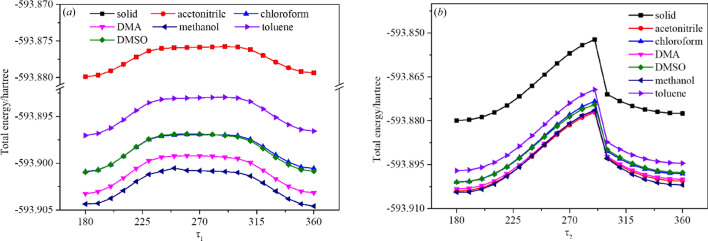
(*a*) Potential energy surface of PHEN τ_1_ in solid and different solvents, (*b*) potential energy surface of PHEN τ_2_ in solid and different solvents.

Conformation adjustment is a vital process during nucleation (Derdour & Skliar, 2014 ▸; Li *et al.*, 2020 ▸). If there is a high rotation barrier between the conformations in solid and solution, conformation adjustment may have an obvious effect on the nucleation process, which could decrease the nucleation rate (Zeglinski *et al.*, 2018 ▸). Thus, a PES about τ_1_ and τ_2_ was generated as shown in Fig. 10 ▸.

From the PES, the results are evident. Regardless of whether it is in solution or solid, the energy change trend with the dihedral angle is the same. Both τ_1_ and τ_2_ reach the minimum energy at 179°, which is the most stable conformation. The conformations in the six solvents are essentially the same as in the solid, indicating they easily transform to the solid conformation during nucleation. Thus, it is reasonable to consider that the conformation adjustment does not have an important effect on the nucleation rate of PHEN in these six solvents.

### 3.8. Molecular interactions   

**Figure 11:**
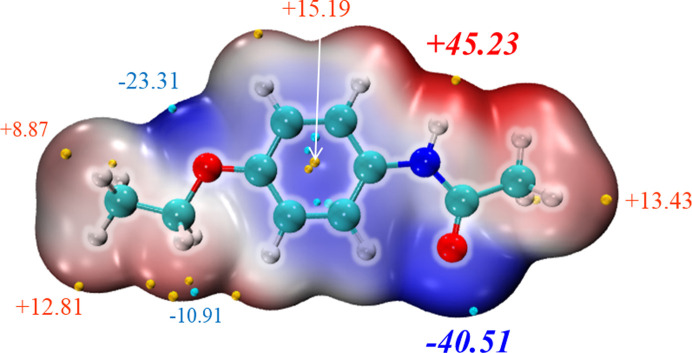
van der Waals surface electrostatic potential of PHEN plotted using *Multiwfn* and *VMD*.

**Figure 12:**
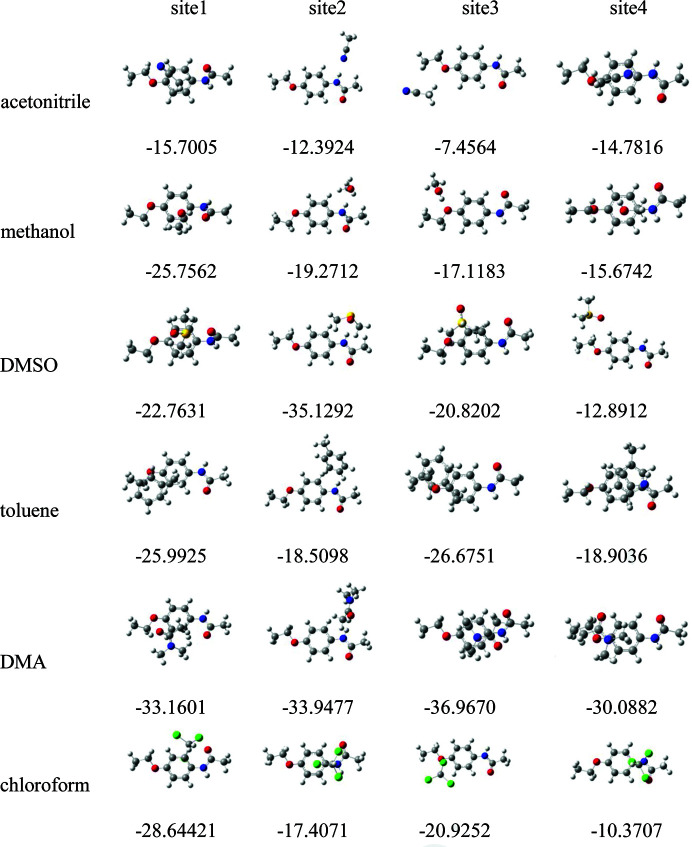
Optimized geometries and binding energies (kJ mol^−1^) for 1:1 PHEN–solvent complexes, calculated at the M062X/6-311G (d,p) level. Carbon – grey, hydrogen – white, oxygen – red, nitro­gen – blue, sulfur – yellow, chlorine – green.

The use of electrostatic potential is a well established approach to predict and explain the relative molecular orientation and the strength of the combination if a complex is mainly assembled by static electricity (such as a hydrogen bond, di­hydrogen bond or halogen bond). And the more negative (or positive) the electrostatic potential is, the more electrophilic (nucleophilic) the atom is likely to be. Therefore, the distribution of the van der Waals surface electrostatic potential of molecules can be analyzed and used to predict the most active sites; four sites on PHEN were selected to optimize the 1:1 solute–solvent complexes and calculate the binding energy. As can be seen from Fig. 11 ▸, the carbonyl oxygen exhibits the greatest electronegativity and was selected as site 1. The amino hydrogen showing the most positive surface electrostatic potential was selected as site 2. The other two sites are eth­oxy oxygen (site 3) and benzene ring π-electrons (site 4). The distributions of the van der Waals surface electrostatic potential of solvent molecules are shown in Fig. S4. The optimized geometries and binding energy results are presented in Fig. 12 ▸.

It can be seen from Fig. 12 ▸ that site 1 presents the largest binding energy in aceto­nitrile, chloro­form and methanol, this is because these all have strong hydrogen-bond donors, favouring the formation of heterodimers which can significantly affect the binding energy. Toluene and DMA as hydrogen-bond donors show the largest binding energy at site 3. Only DMSO as a hydrogen-bond acceptor shows the largest binding energy at site 2.

## 4. Discussion   

Given the above data, it is now possible to investigate the role of solvents in the nucleation process from a molecular perspective. First, the relative nucleation rate of PHEN does not correlate with the fundamental properties of solvents, such as boiling point and density. Also, there is no correlation between the nucleation rate and the relevant solubility.

In recent years, a number of works focused on building a link between the nucleation rate and the solvation strength, such as risperidone (Mealey *et al.*, 2015 ▸), tolbutamide­(Zeglinski *et al.*, 2018 ▸), fenoxycarb (Zeglinski *et al.*, 2019 ▸), salicylic acid (Khamar *et al.*, 2014 ▸) and PABA (Sullivan *et al.*, 2014 ▸). A similar approach was also taken in this study. The nucleation rates followed the order of aceto­nitrile < methanol < DMSO < toluene < DMA < chloro­form, which was consistent with the interface energy. IR and NMR spectroscopy show solute aggregation effects in DMA and chloro­form, but the nucleation rates in chloro­form and DMA are slowest. According to the calculated 1:1 solute–solvent binding energy, the binding energy of site 3 (eth­oxy oxygen) and the nucleation rate are basically consistent, except for chloro­form, indicating that site 3 plays a major role in the nucleation process.

Conformation adjustment also plays an important role in the nucleation process. Thus, the discrepancy in the chloro­form and DMA cases may be caused by a high energy barrier between the conformations in chloro­form and DMA. However, the computational results of the potential energy scan about the torsion angles (τ_1_ and τ_2_) in different solvents demonstrated that neither an obvious conformational difference in chloro­form and DMA nor a high energy barrier exist in this system. Thus, it is concluded that the conformation adjustment should not be the main reason for an abnormal low nucleation rate.

It may be that as the concentration increases, PHEN forms a supramolecular structure in these two solvents which differs from the crystal structure, and then needs to undergo a rearrangement process, so the nucleation rate is slowest. At the same time, the solvation in chloro­form is through a special halogen-bond interaction, which may also cause a relatively large energy barrier to the subsequent rearrangement of the supramolecular structure.

However, when all the solvent systems are considered together, no clear relationship between the specific site interaction, the solvation strength obtained from IR spectroscopy, the conformation structure similarity, the way of gathering and the nucleation difficulty could be summarized. No single factor could individually describe the actual order of the nucleation difficulty and each factor does play a crucial role in certain situations. Therefore, we suggest that none of the four factors: the similarity of the solute in liquid and solid states, the specific site interaction, the way of gathering and the solvation strength, could be neglected. It is speculated that the rearrangement of the supramolecular structure plays a crucial role in determining the nucleation rate.

## 5. Conclusions   

Investigations on the relationship between solution chemistry and nucleation kinetics have been carried out using PHEN as a model compound. We found that form I of PHEN could be obtained in aceto­nitrile, methanol, toluene, chloro­form, DMA and DMSO. The crystal structures were analysed. FTIR, NMR and NOESY spectroscopies were used to analyze the solute species in solution. The results showed that in chloro­form and DMA, PHEN undergoes nucleation through C=O⋯H—N hydrogen bond self-association, whereas in the other solvents PHEN more or less undergoes a desolvation process. However, the nucleation kinetics data showed that chloro­form and DMA had the slowest nucleation rates. We speculate that the self-association in these two solutions produces a supramolecular structure which is different from the solid structure. Rearrangement plays a major role in the rate of nucleation. This work confirms the importance of the self-association and desolvation processes during crystal nucleation. However, since the nucleation of crystals is complicated and many factors could affect the nucleation process, much more work needs to be carried out to fully understand nucleation phenomena.

## Supplementary Material

Supporting information file. DOI: 10.1107/S2052252521003882/lq5039sup1.pdf

## Figures and Tables

**Table 1:** Calculated kinetic and thermodynamic parameters for PHEN nucleation in different solvents

	*A* (m^−3^ s^−1^)	*B* (×10^2^)	*f*_0_ *C*_0_ (m^−3^ s^−1^)	γ (mJ m^−2^)
Aceto­nitrile	17.39	0.22	0.29	0.50
Methanol	10.71	0.76	0.54	0.76
Toluene	18.95	2.23	0.92	1.08
Chloro­form	16.88	4.59	1.32	1.38
DMA	26.81	3.78	1.19	1.29
DMSO	8.55	1.07	0.64	0.85

**Table 2:** Single crystal data of form I

	Form (I)
Empirical formula	C_10_H_13_NO_2_
Formula weight	179.21
Crystal system	Monoclinic
Space group	*P*2_1_/*c*
a (Å)	13.317 (3)
b (Å)	9.6149 (19)
c (Å)	7.7530 (16)
α (°)	90
β (°)	104.05
γ (°)	90
Volume (Å^3^)	963.0 (3)
Density (g cm^−3^)	1.236
*Z*	4
