# Supplementary material for: Unveiling the self-association and desolvation in crystal nucleation
Source: IUCrJ. 2021 Apr 30;8(Pt 3):468–79. doi: 10.1107/S2052252521003882 (PMC8086163; doi:10.1107/S2052252521003882)
Supplement: Supplementary file 1 [file m-08-00468-sup1.pdf]

IUCrJ

Volume 8 (2021)

Supporting information for article:

Unveiling the Self-association and Desolvation in Crystal Nucleation

Danning Li, Yongli Wang, Shuyi Zong, Na Wang, Xin Li, Yuyuan Dong, Ting Wang, Xin Huang and Hongxun Hao

S1. Solubility of PHEN at 25℃ and 40 ℃

Table S1 Solubility of PHEN at 25℃ and 40 ℃

solvent	Solubility at 25°C (g/ml)	Solubility at 40℃ (g/ml)
acetonitrile	0.03589	0.07957
chloroform	0.04923	0.12541
methanol	0.08267	0.16371
toluene	0.00178	0.00420
DMA	0.37864	0.51409
DMSO	0.44140	0.53525

S2. Data of nucleation rates calculated by induction time measured

Table S2 Data of nucleation rates calculated by induction time measured.

Supersaturation S	Average induction time/s	Nucleation rate J/m-3s-1	1/ln2S	ln(J/S)
		acetonitrile		
1.113	370	18.018	87.248	2.784
1.091	490.7	13.320	131.831	2.502
1.077	516	12.503	181.733	2.452
1.057	769.5	8.225	325.414	2.052
1.037	1759	3.531	757.571	1.225
		methanol		
1.171	616	10.210	40.130	2.166
1.150	802	8.313	51.194	1.978
1.127	1023.67	6.385	69.957	1.734
1.106	1208.5	5.304	98.517	1.568
1.085	1615	3.894	150.256	1.278
		toluene		
1.188	586.5	11.367	33.696	2.258
1.165	808	8.089	42.875	1.938

1.143	1018.5	6.294	55.978	1.706
1.121	1771	3.551	76.649	1.153
1.100	3290.5	1.876	110.083	0.534
		chloroform		
1.201	1287	5.013	29.810	1.429
1.186	1448	4.456	34.365	1.324
1.171	2127.5	3.134	40.130	0.984
1.148	4217	1.550	52.494	0.300
1.133	6012	1.073	64.134	-0.054
		DMA		
1.203	593.67	10.594	29.276	2.175
1.180	856	7.788	36.503	1.887
1.157	1110	5.888	47.022	1.627
1.135	1626	3.942	62.360	1.245
1.113	5483.67	1.147	87.248	0.030
		DMSO		
1.170	947.125	7.039	40.568	1.794
1.147	1156.5	5.651	53.163	1.595
1.125	1632	3.928	72.083	1.250
1.104	2042	3.080	102.154	1.026
1.090	2636.5	2.356	134.651	0.771

S3. FTIR of PHEN in chloroform and chloroform-d

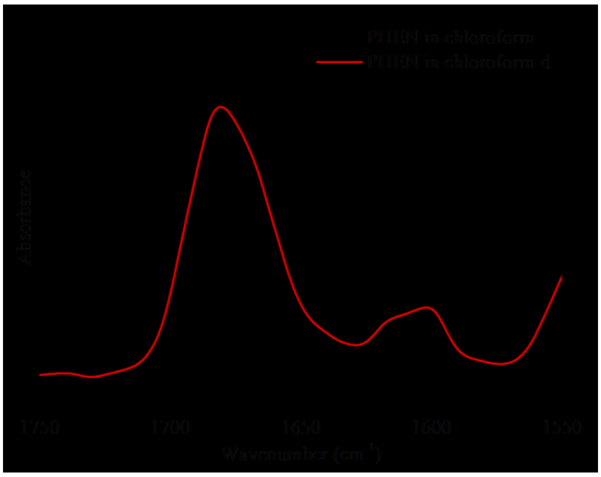

Figure S1 The effect of deuteration of solvent on C=O stretching bands of PHEN in chloroform: the same concentration of PHEN in chloroform and chloroform-d

S4. 2D NOESY of PHEN in acetonitrile-d$_3$, DMSO-d$_6$, and methanol-d$_4$ at room temperature.

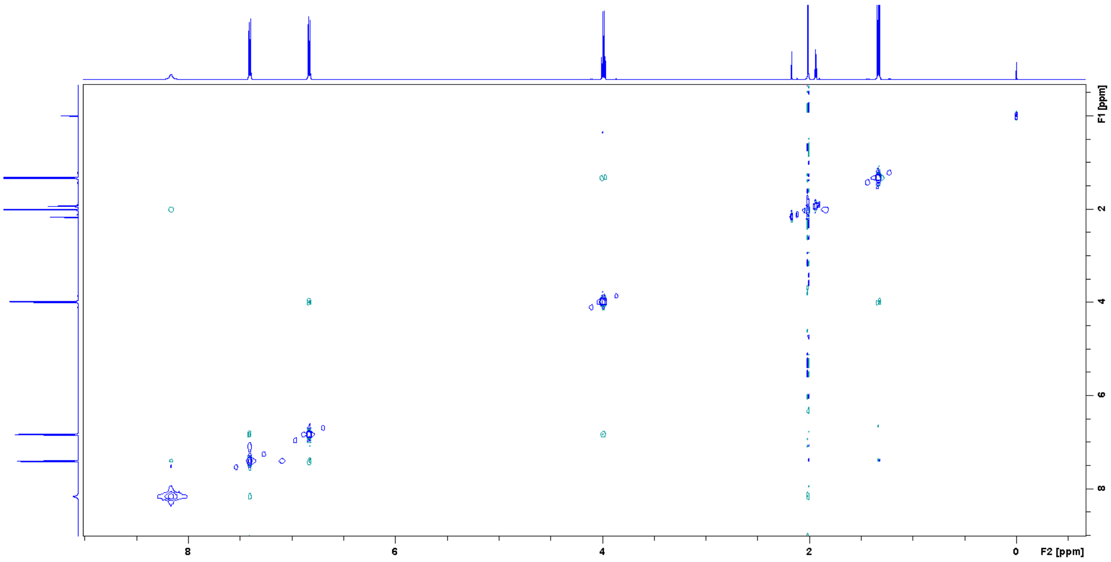

Figure S2 2D NOESY of PHEN in acetonitrile-d$_3$ at room temperature.

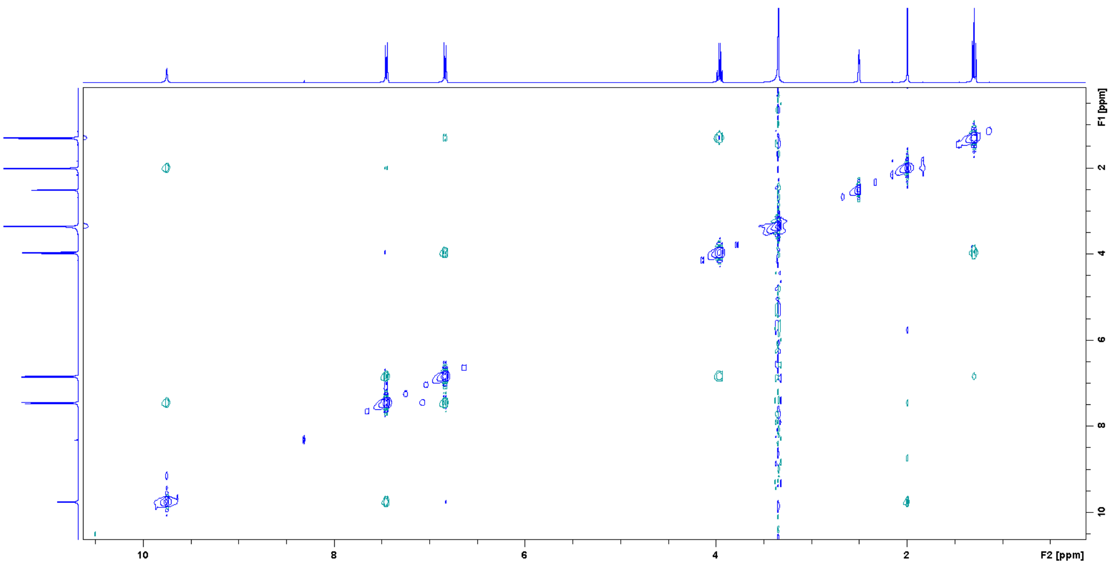

Figure S3 2D NOESY of PHEN in DMSO-d_6 at room temperature.

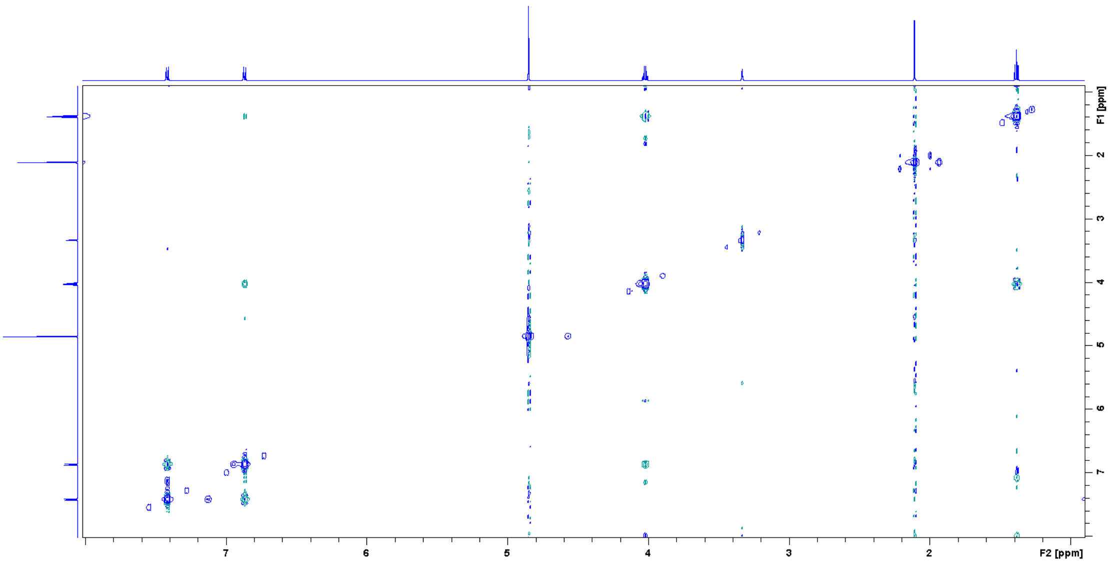

Figure S4 2D NOESY of PHEN in methanol-d_4 at room temperature.

S5. The van der Waals surface electrostatic potential of solvents

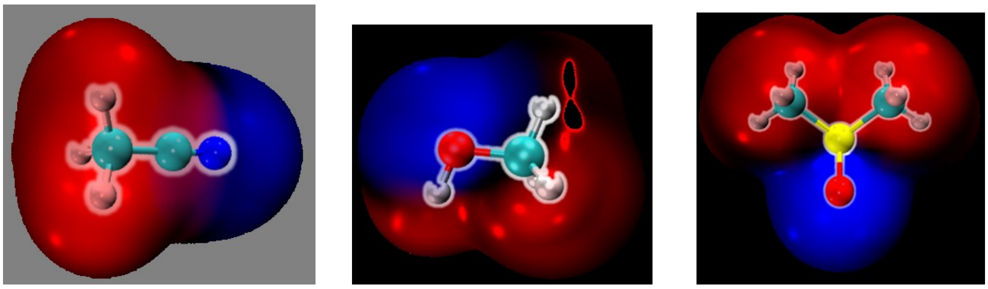

acetonitrile methanol DMSO

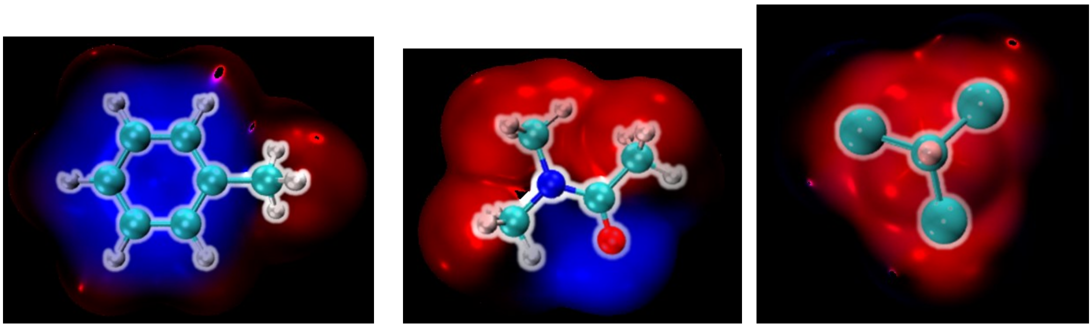

toluene DMA chloroform

Figure S5 The van der Waals surface electrostatic potential of solvent plotted by Multiwfn and VMD
